# Conversion of anti-tissue factor antibody sequences to chimeric antigen receptor and bi-specific T-cell engager format

**DOI:** 10.1007/s00262-024-03778-3

**Published:** 2024-08-06

**Authors:** S. C. Saunderson, J. C. Halpin, G. M. Y. Tan, P. Shrivastava, A. D. McLellan

**Affiliations:** 1 Department of Microbiology and Immunology, University of Otago, Dunedin, 9016 New Zealand; 2 Present Address: The Children’s Hospital Westmead, The Children’s Hospital Westmead CRN Hawksbury Road and Hainsworth Street, Westmead, NSW 2145 Australia; 3 Present Address: Molecular and Clinical Cancer Medicine, The University of Liverpool, Crown St., Liverpool, UK

**Keywords:** Chimeric antigen receptor, Bi-specific T cell engager, Tissue factor, Surface plasmon resonance

## Abstract

**Background:**

The efficacy of antibody-targeted therapy of solid cancers is limited by the lack of consistent tumour-associated antigen expression. However, tumour-associated antigens shared with non-malignant cells may still be targeted using conditionally activated-antibodies, or by chimeric antigen receptor (CAR) T cells or CAR NK cells activated either by the tumour microenvironment or following 'unlocking' via multiple antigen-recognition. In this study, we have focused on tissue factor (TF; CD142), a type I membrane protein present on a range of solid tumours as a basis for future development of conditionally-activated BiTE or CAR T cells. TF is frequently upregulated on multiple solid tumours providing a selective advantage for growth, immune evasion and metastasis, as well as contributing to the pathology of thrombosis via the extrinsic coagulation pathway.

**Methods:**

Two well-characterised anti-TF monoclonal antibodies (mAb) were cloned into expression or transposon vectors to produce single chain (scFv) BiTE for assessment as CAR and CD28-CD3-based CAR or CD3-based BiTE. The affinities of both scFv formats for TF were determined by surface plasmon resonance. Jurkat cell line-based assays were used to confirm the activity of the BiTE or CAR constructs.

**Results:**

The anti-TF mAb hATR-5 and TF8-5G9 mAb were shown to maintain their nanomolar affinities following conversion into a single chain (scFv) format and could be utilised as CD28-CD3-based CAR or CD3-based BiTE format.

**Conclusion:**

Because of the broad expression of TF on a range of solid cancers, anti-TF antibody formats provide a useful addition for the development of conditionally activated biologics for antibody and cellular-based therapy.

**Supplementary Information:**

The online version contains supplementary material available at 10.1007/s00262-024-03778-3.

## Introduction

Immunotherapy of solid cancers is confounded by the lack of reliably expressed tumour-associated antigens and heterogeneity within tumour masses [1]. Chimeric antigen receptor (CAR) T cells have been successfully utilised for the treatment of haematopoietic malignancies, however the expansion into the area of solid tumour treatment has not been as successful [1]. Solid tumours present a number of challenges that prevent effective immunotherapy. In addition to their physical and biological factors, such as the immunosuppressive tumour microenvironment that can lead to the exclusion of effector lymphocytes from the tumour mass, solid tumours are usually heterogeneous in their surface antigen expression [1]. Specific targeting to tumour-associated antigens (TAA) is essential to maximise anti-cancer effects while avoiding systemic toxicities [2, 3]. Immunotherapy of solid tumours face a number of challenges including the immunosuppressive tumour environment, and low level TAA expression on some non-malignant tissues leading to on-target off-tumour effects [4, 5].

Antibody sequences form the recognition domain of clinically approved therapies for blood cancers including bi-specific T-cell engagers (BiTEs) and CAR T cells. All FDA-approved CAR possess an N-terminal scFv domain, although other experimental anti-tumour ligands have been proposed [6, 7]. BITEs target T cell activation molecules (e.g. the CD3 complex) together with an antigen expressed on the surface of on tumour cells. The tethering of T cells to cancer cells provokes potent T cell activation and destruction of tumour cells.

CAR T cells are engineered with surface expressed immunoglobulin-derived single chain variable fragments (scFv) linked to intracellular T cell signalling domains such as CD3, CD28, or 41BB, thus enabling T cells to directly target cells expressing a specific TAA expressed on tumour cells [8, 9]. Targeting more than one antigen can expand tumour recognition and mitigate immune escape through loss of the expression of a single antigen. Requiring dual recognition can also improve safety. Alternatively, off-switches can be used to prevent unwanted reaction to non-malignant tissue. For example, split CAR designs require recognition of two TAA for full T cell activation due to the presence of a singular intracellular activation domain on each CAR. While bi-specific T cell engager (BiTE) molecules that link two distinct scFv together, one specific for T cells, e.g. anti-CD3 on CAR T cells or endogenous tumour infiltrating T cells (TILs), and the other towards an antigen of choice, e.g. a TAA [10].

In order to expand the possibilities of targeting solid tumour-associated TAA, we investigated well-characterised anti-tissue factor monoclonal antibodies (mAb) for conversion into CAR or as a bi-specific T cell engager utilising and anti-CD3 gamma/epsilon BiTE arm as a proof of concept using an antigen widely expressed on solid tumours and one that is associated with poor prognosis. Tissue factor (TF; CD142) is highly overexpressed on many solid tumours, such as pancreatic and triple-negative breast cancers, at levels 1000-fold higher than normal tissue [11–13]. This also includes expression within the neo-vasculature of the tumour, overall making TF a potentially useful TAA to target in tumour immunotherapy [11, 13–17]. Dysregulation of gene expression due to loss of tumour suppression results in over-expression of TF in a number of cancers [11, 18, 19]. TF expression in tumour cells is linked to the mutations in p53 and phosphatase and tensin homolog PTEN [19], resulting in dysregulation of TF expression. TF expression enhances cancer progression and metastasis, not only via coagulation and platelet activation but also by TF activation of protease activated receptor-2 (PAR-2) signalling [11, 19–25].

TF binds to circulating coagulation factor VIIa (FVIIa) to form a TF and FVIIa catalytic complex, initiating the extrinsic coagulation pathway and resulting rapid and profound coagulation, often associated with pathological events, such as venous thromboembolism. We have previously shown that exogenous Factor V binds to tumour-associated TF, particularly when the TF is associated with phosphatidylserine-exposed tumour-membranes and vesicles [26, 27]. The TF coagulation factor is absent from haematopoietic cells, but is expressed as part of the haemostatic envelope on cells surrounding the vasculature [18], primarily in subendothelial cells which anatomically sequester TF from the blood [28–30]. While this reduces the interaction between CAR T cells and non-malignant cells expressing TF, in turn decreasing on-target off-tumour effects, there are still reports of low level TF expression within the body. TF targeting therefore carries the potential to cause life-threatening on-target off-tumour affects, further emphasising the use of a dual recognition immunotherapy system [30].

In this study, we have investigated the ability to express two well-known anti-human TF mAb (clones TF8-5G9 and hATR-5) as scFvFc and confirm their binding affinity to TF. Further to this we developed two functional 2nd generation CAR constructs derived from TF8-5G9 and hATR-5 scFv. However, as solid tumour immunotherapies targeting a single antigen run a greater risk of life-threatening on-target off-tumour effects, it may be prudent to employ a second antigen or target to further reduce risks [2]. We therefore generated an anti-TF/-CD3 BiTE from the TF8-5G9 and OKT3 scFv which successfully targeted both the TF tumour cell and CD3 of T cells. This study has further emphasised the immunotherapeutic potential of targeting TF expressed by tumour cells.

## Materials and methods

### Cell culture

Jurkat (clone E6.1) and MDA-MB-231, or HEK293T were cultured respectively at 37 °C with 5% CO_2_ in RPMI media supplemented with 5% foetal calf serum (FCS; Pan Biotech), 55 µM β-mercaptoethanol, 100 U/mL penicillin and 100 µg/mL streptomycin (unless otherwise stated), or 10% FCS/DMEM media supplemented with 100 U/mL penicillin and 100 µg/mL streptomycin (unless otherwise stated). Expi293F cells were cultured at 37 °C with 8% CO_2_ and orbital shaking (130 rpm) in Expi293F expression media (ThermoFisher).

### Constructs

Codon optimised anti-human TF scFv sequences were derived from the amino acid sequences of murine mAb clone TF8-5G9 (PDB: 1FGN; [31, 32]) and the murine-humanised mAb clone hATR-5 (PDB: 1UJ3; [33]). Plasmid insert gene constructs and DNA sequences are listed in supplementary data.

### Protein expression and purification

Planktonic Expi293F cells (37.5 × 10^6^ in 15 mL) were transfected with 15 µg of pcDNA3.1(-):TF85G9 scFvFc, hATR-5 scFvFc, or human TF-Fc (Genscript), or pSBbiGP:OKT3-TF85G9 BiTE (His-tagged; OKT3 VH-VL and TF85G9 VH-VL joined via glycine-serine linkers; IDT geneblock) using the Gibco Expifectamine293 transfection kit as per the manufacturer’s instructions (Gibco #A14524). Cells were incubated until cell viability decreased to approximately 30%, after which cell supernatant was centrifuged at 450 × g for five minutes, followed by 2000 × g for 20 min, and supernatant 0.2 µm filtered. Fc-fusion proteins were purified by Protein A chromatography (Pierce #20,356), eluted in 500 µL fractions of 100 mM glycine (pH 3.0) and neutralised with 100 µL of 1 M Tris–HCl (pH 9.0). His-tagged proteins were purified by Protino Nickel-NTA agarose column (MN745400.25) as per the manufacturer’s instructions, prior to dialysis with phosphate-buffered saline (PBS). For SPR, fusion proteins were further purified by size exclusion chromatography (Superdex 200 10/300 GL column) with PBS-EP (0.01% Tween20, 300 mM NaCl PBS, pH 7.4). In brief, proteins were centrifuged at 15,000 × g to remove precipitate, followed by incubation with 2 mM DTT for 30 min on ice. Proteins were then injected in the column and run at a flow rate of 0.5 mL/min and 500 µL fractions collected. Boiled and reduced proteins were run on a 4–12% BOLT Bis–Tris gel, before stained with Coomassie blue.

### SPR

SPR experiments were carried out using a Biacore X100 instrument at 25 °C. Human TF-Fc was immobilised to the surface of a CM5 sensor chip (target of 500 response units) in 10 mM sodium acetate pH 4.0 with a flow rate of 10 µL/min. Serial dilutions (18.75–3.125 nM) of TF8-5G9 scFvFc were made with PBS-EP and run over the sensor chip for two cycles (flow rate 30 µL/min; contact time 180 s; dissociation time 600 s). Sensograms were globally fitted with a 1:1 binding model and binding kinetics determined with BIAevaluation software.

### CAR generation

Second-generation CAR constructs (scFv-c-myc tag-CD8 hinge-CD28-CD3ζ) were generated from the variable fragment sequences of anti-human tissue factor mAb TF8-5G9 and hATR-5. A sleeping beauty transposon system was utilised for transfection of the CAR cassette into Jurkat T cells. Plasmids (9 µg of pSBbiGP (or RP where stated) and 1 µg of pCMV(CAT)T7-SB100) were introduced into cells via electroporation with a neon transfection system with 100 µL gold tips (1350 V, 10 ms, three pulses) and cultured with antibiotic free media for at least 48 h, before stably transfected cells selected for with 2 µg/mL puromycin. TF8-5G9 or hATR-5 CAR T cells were incubated with 2 µg/mL anti-c-myc-biotin (Biolegend #908,805) in 0.1% BSA/PBS/2 mM EDTA for 30 min on ice, biotin was then detected with 4 µg/mL streptavidin-allophycocyanin (Biolegend #405,207) and incubated as previously described. Cells were analysed for GFP or RFP (as stated) and c-myc expression with a BD LSR Fortessa, and subject to both FSc and SSc doublet discrimination prior to further analysis with FlowJo v10.7.2.

### In vitro CAR assay

A 24-well plate was seeded with TF-expressing cell line MDA-MB-231 (1 × 10^6^ cells/mL; 500 μL/well) in 5% FCS/RPMI for 24 h at 37 °C with 5% CO_2_. Alternatively, wells were coated with human TF-Fc fusion protein (10–1.25 µg/mL; 300 μL/well) overnight at 4 °C, wells were then rinsed twice with PBS. CAR-T or untransfected Jurkat cells (1 × 10^6^ cells/mL; 600 μL/well) in 5% FCS/RPMI were then added to the plates and incubated at 37 °C with 5% CO_2_ for 24 h. Positive control 25 ng/mL PMA/0.75 µg/mL ionomycin, or negative controls as stated were also included. Cell culture supernatant was analysed by anti-human IL-2 ELISA.

### In vitro BiTE assay

HEK293T cells (2 × 10^5^) were seeded in 24-well plates in 500 μL of antibiotic-free media and incubated at 37 °C with 5% CO_2_. At 24 h, HEK293T were transiently transfected with a total of 500 ng of plasmid DNA (pcDNA3.1( +): Human full-length TF, pSBbiGP: OKT3-TF85G9 BiTE, pSBbiGP: empty vector, or a 1:1 ratio of two plasmids where stated) using lipofectamine 3000 as described by the manufacturer. Media was changed and Jurkat cells (1 × 10^6^ cells/mL; 500 μL) were added in 10% FCS/RPMI at 16 h post-transfection. Positive control 25 ng/mL PMA/0.75 µg/mL ionomycin, or negative controls R10 alone. Cells were further incubated at 37 °C with 5% CO_2_ for 24 h before culture supernatant was removed for IL-2 analysis.

### IL-2 ELISA

Capture antibody anti-human IL-2 (2 µg/mL; BD #555,051) in PBS was coated overnight at 4 °C. Wells were washed three times with 0.05% Tween20/PBS, prior to blocking with 1% BSA/PBS for ten minutes at room temperature and blocking solution flicked out. An IL-2 standard or cell supernatant was added to the wells and incubated overnight at 4 °C. Wells were washed as previously described and incubated with biotinylated anti-human IL-2 (1 µg/mL; BD #555,040) diluted in 1% BSA/PBS for one hour at 37 °C. After washing, wells were incubated with streptavidin horse radish peroxidase (1-in-5000 dilution; Roche #11,089,153,001) for one hour at 37 °C. Wells were washed and developed in 3,3′,5,5;-tetramethylbenzidine (Novex #00–2023), the reaction was then stopped by the addition of 2N H_2_SO_4_ and the plate read at 450 nm.

## Results

**Fig. 1:**
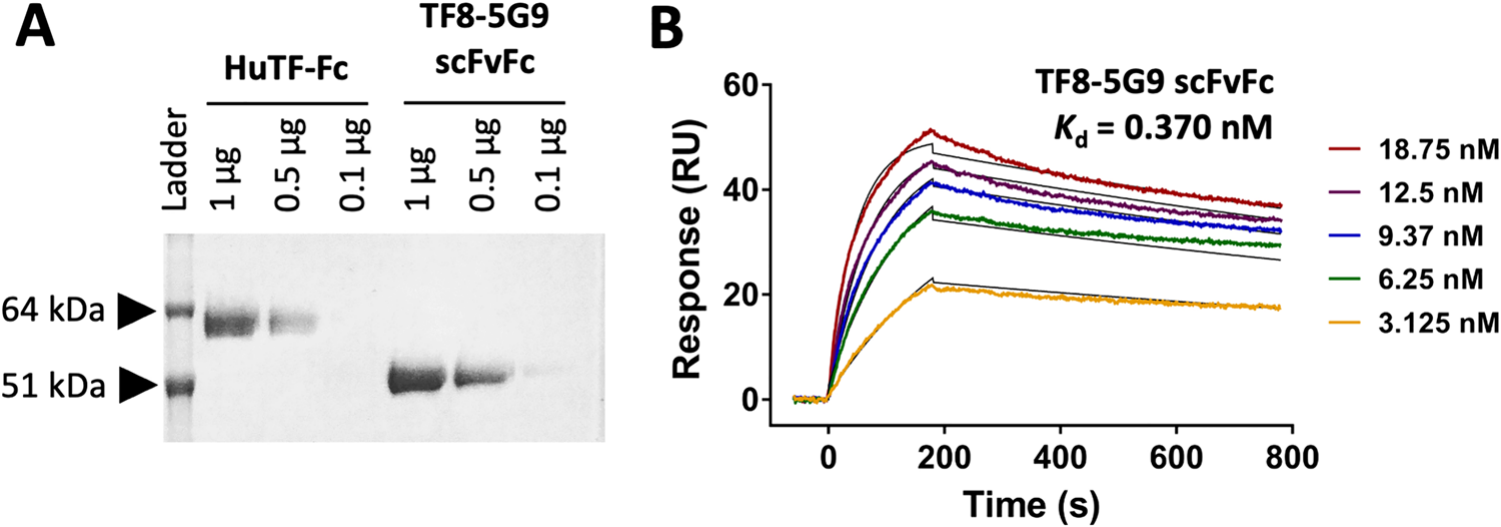
TF8-5G9 anti-TF scFvFc binding affinity for human TF-Fc. Expi293F cells were transfected with pcDNA3.1(-) plasmids containing either TF8-5G9 scFvFc or human TF-Fc. Cell culture supernatant was harvested and Fc fusion protein purified via Protein A affinity chromatography column, followed by size exclusion chromatography. **A** TF8-5G9 anti-TF scFvFc and human TF-Fc (boiled and reduced; 1000, 500 and 100 ng) were run on an SDS-PAGE gel and Coomassie blue stained. Gel result representative of two independent Fc-fusion protein preparations. **B** Purified human TF-Fc antigen was immobilised by amine-coupling to CM5 Biacore sensor chips. A titration of purified TF8-5G9 scFvFc was allowed to bind for a contact time of 180 s at a flow rate of 30 µL/min, followed by a dissociation time of 600 s with a Biacore X100 SPR machine at pH 7.4. Titration curves in descending order: 18.75, 12.5, 9.375, 6.25 and 3.125 nM. SPR results representative of two repeats

A number of solid tumours express high TF levels [11, 13–16], we therefore aimed to test the utility of known anti-human TF monoclonal antibodies in the context of a CAR T cell or BiTE. To ensure the binding capacity of TF8-5G9 was not lost when the variable regions of this mAb were expressed as a scFvFc construct [32], we expressed the TF8-5G9 scFv and its antigen human TF as Fc fusion proteins (Fig. 1A). TF8-5G9 retained a high binding affinity (*K*_d_ = 0.37 nM) as determined by SPR (Fig. 1B).

**Fig. 2:**
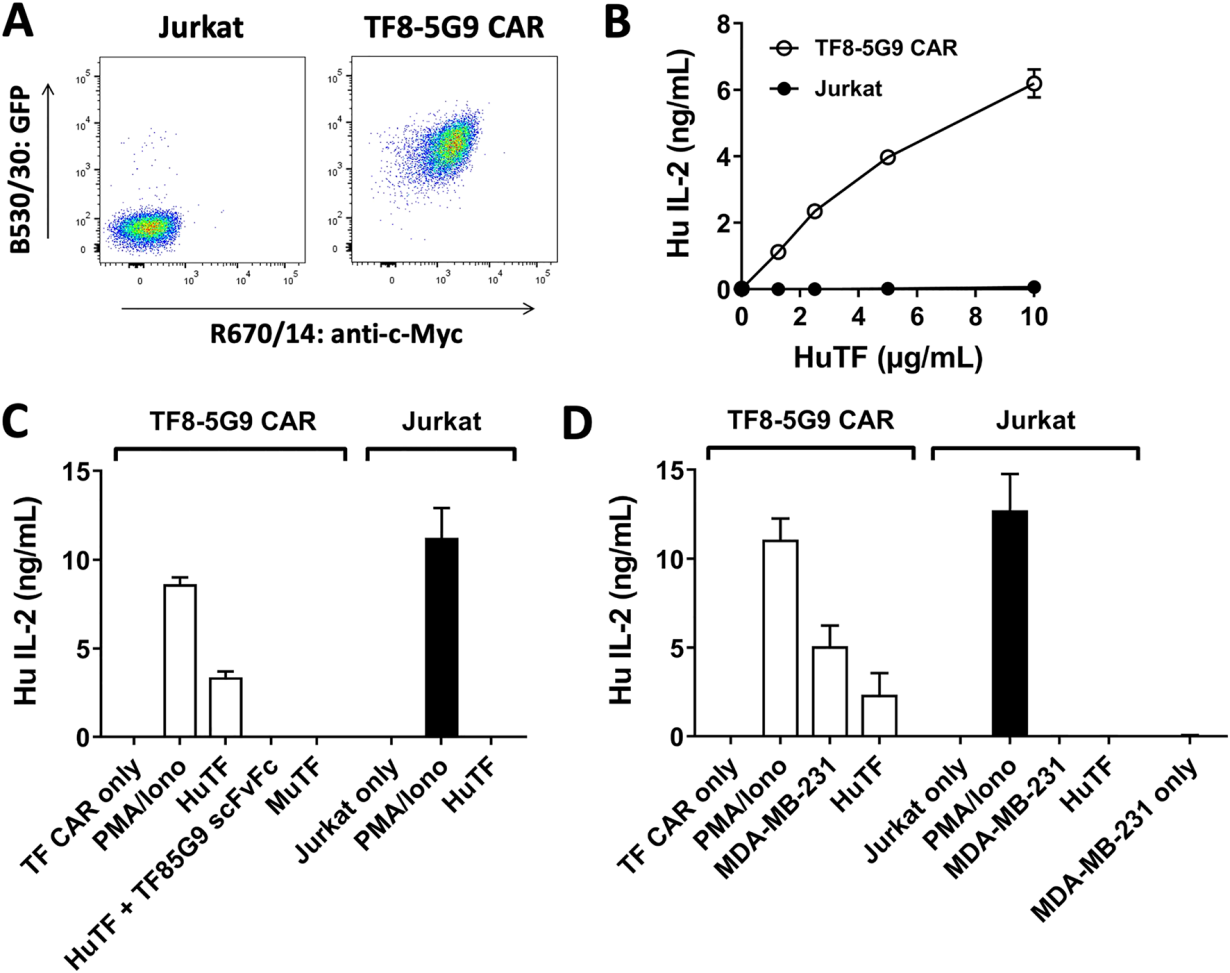
TF8-5G9 anti-TF CAR is specifically activated by human TF in vitro. **A** TF8-5G9 CAR-T or untransfected Jurkat cells were analysed for c-Myc and GFP expression by flow cytometry. IL-2 release from TF8-5G9 CAR-T or untransfected Jurkat was measured by ELISA following incubation for 24 h with **B** a titration of solid phase human TF, **C** negative controls nil antigen, murine TF or competitive inhibition with TF8-5G9 scFvFc, or **D** TF-expressing MDA-MB-231 cells, where stated positive controls PMA/ionomycin or 2.5 µg/mL solid phase human TF were included. Results representative of three independent repeats, **B** a single pilot experiment where points indicate mean ± SD of triplicate wells, and **C** and **D** bars indicate mean with SD error bars of three pooled independent experiments

We next constructed a second-generation CD3/CD28 CAR with c-Myc tag by utilising the TF8-5G9 scFv. This was cloned into a sleeping beauty transposon system with SfiI restriction sites [34], prior to its transfection into the Jurkat (Clone E6.1) cell line that is known to express high levels of IL-2 upon CD3/CD28 signalling. Successful transfection and expression were observed via the upregulation of the surrogate marker GFP, as well as the c-Myc tag within the CAR construct (Fig. 2A).

The TF8-5G9 CAR showed a dose-dependent response upon exposure to HuTF with increasing levels of IL-2 detected (Fig. 2B). The TF8-5G9 CAR response could be neutralised by the inclusion of TF8-5G9 scFvFc emphasising the CAR’s specificity. Furthermore, no cross-species reactivity was noted in the presence of murine TF (Fig. 2C). Triple negative breast cancer cell line MDA-MB-231 has previously been shown to express TF [13], therefore we tested the CAR T cell capacity to recognise endogenous levels of TF. A clear response was observed with approximately 5 ng/mL IL-2 detected following incubation of TF8-5G9 CAR T cells with MDA-MB-231 (Fig. 2D).

**Fig. 3:**
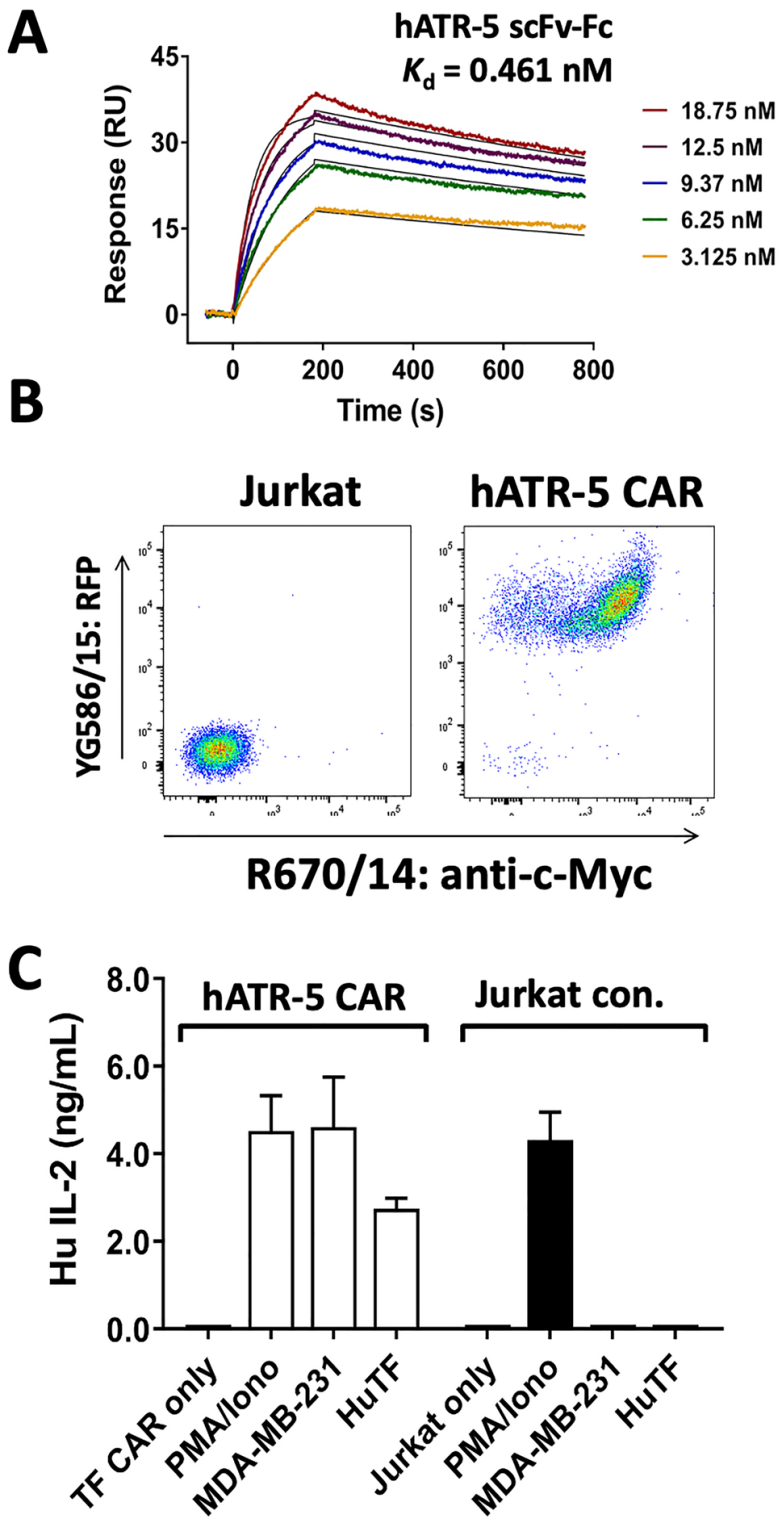
hATR-5 anti-TF CAR targets TF-expressing tumour cell line in vitro. **A** Purified human TF-Fc antigen was immobilised by amine-coupling to CM5 Biacore sensor chips. A titration of purified hATR-5 scFvFc was allowed to bind for a contact time of 180 s at a flow rate of 30 µL/min, followed by a dissociation time of 600 s with a Biacore X100 SPR machine at pH 7.4. Titration curves in descending order: 18.75, 12.5, 9.375, 6.25 and 3.125 nM. SPR results representative of two repeats. **B** hATR CAR-T or untransfected Jurkat cells were analysed for c-Myc and RFP expression by flow cytometry. **C** IL-2 release from hATR-5 CAR-T or untransfected Jurkat was measured by ELISA following incubation for 24 h with TF-expressing MDA-MB-231 cells, 2.5 μg/mL solid phase human TF, cell only controls or positive control PMA/ionomycin. Results representative of two independent experiments. Bar indicates mean with SD error bars

In a similar manner to the TF8-5G9 CAR construct, we investigated the use of a second anti-human TF monoclonal antibody, hATR-5 [33]. hATR-5 scFv was found to have a high binding affinity (*K*_d_ = 0.46 nM) by SPR (Fig. 3A). The hATR-5 scFv when incorporated into a CAR construct was both surface expressed as shown by flow cytometric analysis of c-Myc expression, as well as functional, with a clear response against both solid-phase TF, as well as endogenously expressed TF on MDA-MB-231with IL-2 detected by ELISA (Fig. 3B and C).

**Fig. 4:**
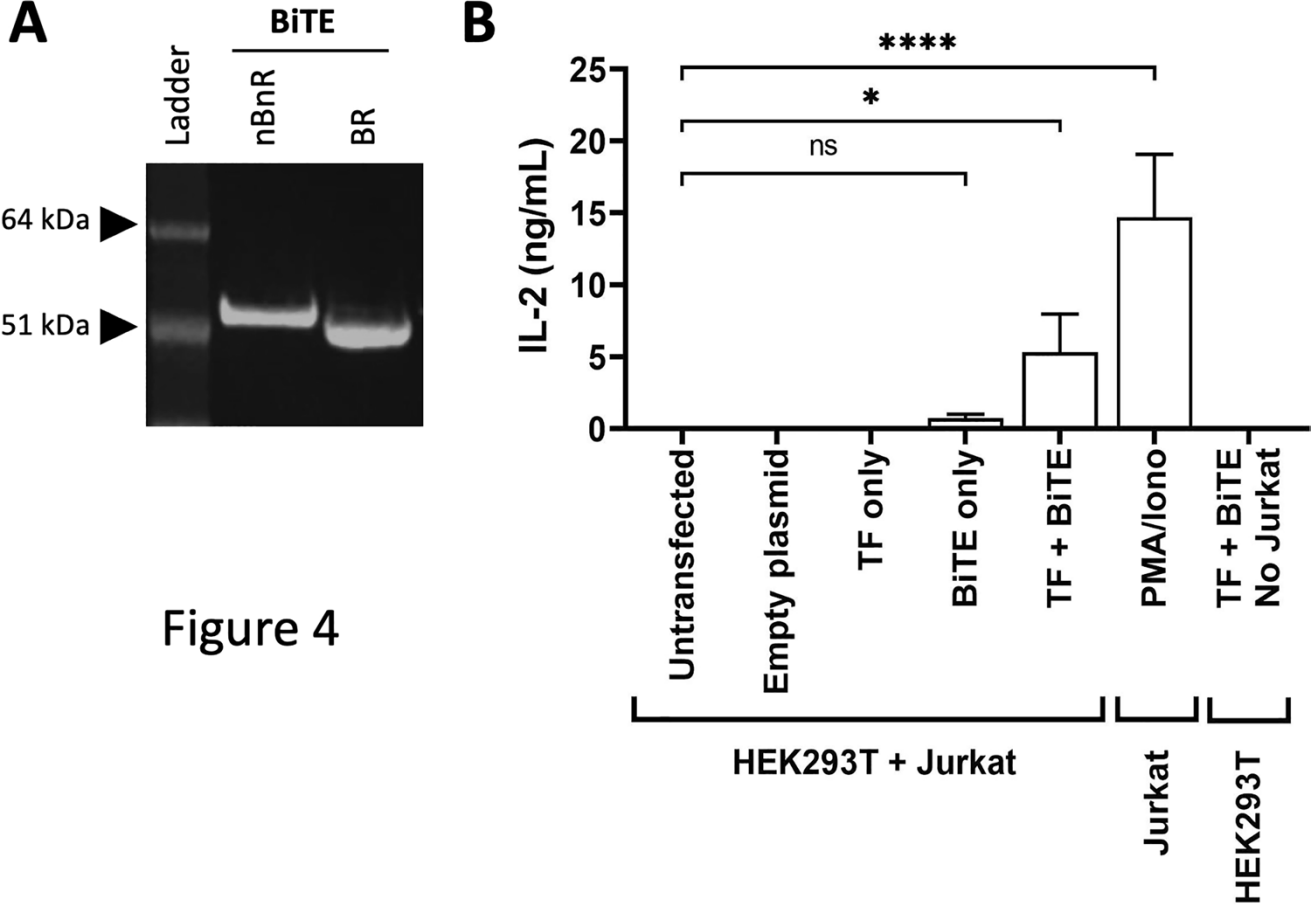
Anti-TF-anti-CD3 BiTE activates T cells in the presence of TF-expressing cells in vitro*.* **A** Expi293F cells were transfected with pSBbi-GP plasmid containing OKT3-TF85G9 BiTE. His-tagged protein was purified from cell culture supernatant via a Nickel-NTA column. OKT3-TF85G9 BiTE protein either not boiled/not reduced (nBnR) or boiled/reduced (BR) were run on an SDS-PAGE gel and Coomassie blue stained. Gel representative of at least two preparations. **B** HEK293T cells were transiently transfected or co-transfected with plasmids containing either human TF, anti-TF-anti-CD3 BiTE, or an empty vector as stated. At 16 h post-transfection, Jurkat T cells were added and further incubated for 24 h before IL-2 release was analysed by ELISA. Untransfected HEK293T with Jurkat cells or co-transfected TF/BiTE without Jurkat cells were used as additional negative controls, and PMA/ionomycin as a positive control. Bar indicates mean with SD error bars of three pooled independent experiments. One-way ANOVA with Bonferroni post-correction test performed: ns = not significant; * *P* < 0.05; **** *P* < 0.0001

To investigate the potential of utilising TF within a dual target system, we generated a BiTE that binds both human TF (TF8-5G9) and CD3 (OKT3) [35]. This BiTE was soluble and able to be purified from Expi293F secreting cells, however corresponding with previous studies a low yield indicated relatively low expression (data not shown). HEK293T cells were transiently transfected to express both the full-length TF antigen, as well as the anti-TF/CD3 BiTE. In the presence of Jurkat T cells, IL-2 was released indicating that the anti-TF/CD3 BiTE was functional (Fig. 4).

## Discussion

In this study, we developed CAR and BiTE from well-characterised monoclonal TF sequences, clones TF8-5G9 and hATR-5 [32, 33, 36]. For both antibodies, we demonstrated a preservation of TF binding affinity following conversion to scFv format (*K*_d_ 0.37 and 0.46 nM respectively; Fig. 1B and 2A). Previous reports of TF8-5G9 and hATR-5 Fab has shown high affinity binding (3.4 nM (*K*_i_), and 0.85 nM) to the C-terminal side of the TF extracellular domain [33, 36]. This high affinity is largely contributed by the presence of 15 and 19 hydrogen bonds within the heavy and light chain CDRs of the TF8-5G9 and hATR-5 clones, respectively [33, 36].

While the development of a scFv into a CAR T cell can be a straightforward conversion, this is not always the case. scFv-derived CAR do not always show sufficient surface expression due to defective protein synthesis and/or inefficient transport to the cell surface. While, some scFv-derived CAR may show high surface expression but lack binding functionality due to the aggregation of CAR on the cell surface, or poor structural stability once cell surface-expressed [37]. In contrast, CDR-mediated CAR clustering has been shown to cause antigen-independent tonic signalling within CAR-T cells leading to T cell exhaustion [38]. We demonstrated the successful utility of anti-TF mAb constructs from the clones TF8-5G9 and hATR-5 for incorporation into a CAR format, as well as developing the first anti-TF BiTE bridging TF and CD3 on T cells (Figs. 2, 3 and 4).

The success of cancer immunotherapy is largely reliant on the TAA selected to target. Challenges arise when TAA are expressed elsewhere within the body. Both anti-TF CAR were surface expressed on human T cells and showed dose dependent cytokine release in response to solid-phase or cell-associated TF antigen. Similarly, the TF8-5G9 BiTE was expressed in a mammalian system and demonstrated the BiTE developed reacted specifically to TF. On-target off-tumour effects could be addressed by utilising conditional reactivity. This can be achieved by a variety of means including affinity detuning, mutation and masking to allow selective activation in the tumour environment. As part of our characterisation, we determined that both anti-TF scFvFc TF8-5G9 and hATR-5 have nanomolar binding affinities, as such there is greater flexibility to potentially reduce the binding affinity via rational or random mutagenesis to achieve micromolar binding affinities [39]. Where CAR still retain potent anti-tumour activity, while exhibiting superior safety profiles by sparing tissues with low antigen density, e.g. self-tissue as only high density antigen is able to trigger the CAR T cells [39–41].

The utilisation of dual recognition immunotherapies, e.g. BiTEs have the potential to not only improve the safety profile of TF-targeted cancer therapies by only activating T cells in the tumour vicinity. But have the added advantage of enhancing both CAR T cells, and/or endogenous TIL activation. Pre-clinical studies with BiTE AMG 596 targeting an anti-EGFRvIII tumour antigen and CD3 has shown promising results with prolonged survival of tumour bearing mice in vivo, thus demonstrating the clinical utility of BiTE technology [42]. Previous studies have shown promising results by the targeting of TF in a variety of approaches, including monoclonal antibody blockade, chimeric antigen receptor expressing cells, or modified anti-TF molecules. Tisotumab Vedotin, an anti-TF mAb (TF-011) drug conjugate has shown promising anti-tumour activity in clinical trials and is currently FDA-approved for recurrent and metastatic cervical cancer [43, 44]. Zhu et al. utilised TF-011 in an alternative context with the construction of a T cell engaging bispecific antibody (TCB) which utilised TF-011 light chains linked by their C-termini to anti-CD3 (OKT3) scFv [45]*.* They showed significant anti-tumour activity both in vitro and in vivo with the anti-TF/CD3 TCB. Alternatively, while traditional CAR utilise an immunoglobulin-derived scFv fragment, a promising non-traditional 3rd generation anti-TF CAR T cell was created utilising the Factor VII light chain rather than a scFv [46].

Doxorubicin-encapsulated liposomes were effectively targeted to both TF-expressing tumour cells via the use of anti-TF Fab fragments PEG-conjugated to liposomes. Tumour-bearing mice showed both reduced tumour growth and increased survival when treated with these liposomes. Moreover, the importance of targeting both the tumour directly, in addition to surrounding TF-rich stromal cells within the tumour microenvironment was shown via the use of liposomes conjugated to both anti-human and anti-murine TF antibodies, where pancreatic tumour cells (BxPC3) of human origin were xenografted into mice, while the stromal cells of the tumour microenvironment were of murine origin [47]. Overall, a diverse range of immunotherapies utilising anti-TF mAb to target tumours are currently being researched and show promise, thus the addition of new sequences to the library of successfully expressed and functional anti-TF scFv will further aid research.

A limitation of this study is that the CAR was only tested in vitro. Future work will be required to determine if anti-TF CAR and BiTE will be effective against solid tumours in a preclinical model. Because the TF8-5G9 anti-TF CAR showed no reaction to murine TF, this would allow in vivo mouse experiments to performed without concern of any on-target off-tumour effects of using this particular scFv clone. However, to fully determine safety of such a construct, backcrossing of the huTF allele from existing lines into NSG mice would be required for adoptive transfer of human CAR T cells and testing of human tumour cell lines [48].

This work provides a template for the future development of anti-TF agents to target solid tumours. A primary requirement for TF-targeting strategies will be enforcing conditional activity of such agents specifically at the tumour site. Therefore, the CAR and BiTE formats developed here are a first step towards incorporation into a conditional or inducible activation logic.

## Supplementary Information

Below is the link to the electronic supplementary material.

Supplementary file1 (DOCX 19 kb)

## Data Availability

No datasets were generated or analysed during the current study.
