# Supplementary material for: Conversion of anti-tissue factor antibody sequences to chimeric antigen receptor and bi-specific T-cell engager format
Source: Cancer Immunol Immunother. 2024 Aug 6;73(10):195. doi: 10.1007/s00262-024-03778-3 (PMC11303627; doi:10.1007/s00262-024-03778-3)
Supplement: Supplementary file 1 — Supplementary file1 (DOCX 19 kb) [file 262_2024_3778_MOESM1_ESM.docx]

**Conversion of anti-tissue factor antibody sequences to chimeric antigen receptor and bi-specific T cell engager format.**

^1^Saunderson SC, ^1^Halpin JC, ^1^Tan GMY, ^1^Shrivastava P, ^1^*McLellan AD

^1^Department of Microbiology & Immunology, University of Otago, Dunedin, New Zealand.

*Address correspondence to: Prof. Alexander McLellan; Department of Microbiology and Immunology, University of Otago, Dunedin 9016, New Zealand E-mail: alex.mclellan@otago.ac.nz

**Construct design**

pcDNA3.1(-): TF8-5G9 / hATR-5 scFvFc: SS – VH – GS linker – VL – IgG1 Hinge – CH2 – CH3

pSBbi-RP hATR CAR: SS – hATR-5 VH – GS linker – hATR VL – c-myc tag – Hu CD8 hinge – Hu CD28 TM – Hu CD28 cytoplasmic – Hu CD3 zeta

pSBbi-GP TF8-5G9 CAR: SS – TF8-5G9 VH – GS linker – TF8-5G9 VL – c-myc tag – Hu CD8 hinge – Hu CD28 TM – Hu CD28 cytoplasmic – Hu CD3 zeta

pcDNA3.1(-) TF-Fc: SS – sHuTF – IgG1 Hinge – CH2 – CH3 – OctHis

pcDNA3.1(-): SS – TF8 LC – (G4S1)3 linker – TF8-5G9 HC – (G3S1)1 linker – OKT3 HC – (G2S1)4 linker – OKT3 LC – c-myc – Oct-His

**hATR-5 scFvFc sequence**

ATGGATTTTCAGGTGCAGATTTTCAGCTTCCTGCTAATCAGTGCCTCAGTCATAATGTCTGCTCAAGTCCAGCTCCTCGAAAGCGGGGCTGTACTTGCTCGGCCAGGAACCAGTGTAAAAATATCATGTAAGGCGTCTGGATTCAATATAAAGGATTACTATATGCACTGGGTTAAACAAAGGCCCGGCCAAGGATTGGAATGGATAGGAGGCAACGATCCCGCCAACGGTCATTCTATGTATGATCCTAAATTTCAAGGGCGCGTTACAATTACAGCCGATACATCAACTAGCACCGTGTTTATGGAGCTCTCATCCCTCAGATCTGAAGACACCGCCGTATATTATTGTGCTCGAGACAGTGGTTATGCAATGGATTATTGGGGTCAGGGTACTCTTGTCACCGTTAGCAGCGGCGGTGGCGGTTCTGGTGGCGGTGGCTCCGGCGGTGGCGGTTCTGACATTCAGATGACTCAATCTCCTTCAAGTCTTTCTGCTAGTGTAGGTGACAGGGTGACGATAACGTGCAAGGCGAGCCAAGATATAAAGTCTTTCCTGTCTTGGTATCAGCAGAAGCCCGAGAAAGCTCCCAAGAGCTTGATATACTACGCCACGTCTCTCGCAGACGGAGTTCCCTCTCGCTTTTCTGGGTCTGGGTCCGGTACAGACTACACCCTGACGATTAGTTCACTTCAACCGGAAGATTTTGCTACCTACTACTGTCTTCAGCACGGGGAGTCTCCATATACATTTGGGGGTGGGACTAAAGTAGAGATAAAACGCGGTACCGACAAAACTCACACATGCCCACCGTGCCCAGCACCTGAACTCCTGGGGGGACCGTCAGTCTTCCTCTTCCCCCCAAAACCCAAGGACACCCTCATGATCTCCCGGACCCCTGAGGTCACATGCGTGGTGGTGGACGTGAGCCACGAAGACCCTGAGGTCAAGTTCAACTGGTACGTGGACGGCGTGGAGGTGCATAATGCCAAGACAAAGCCGCGGGAGGAGCAGTACAACAGCACGTACCGTGTGGTCAGCGTCCTCACCGTCCTGCACCAGGACTGGCTGAATGGCAAGGAGTACAAGTGCAAGGTCTCCAACAAAGCCCTCCCAGCCCCCATCGAGAAAACCATCTCCAAAGCCAAAGGGCAGCCCCGAGAACCACAGGTGTACACCCTGCCCCCATCCCGGGAGGAGATGACCAAGAACCAGGTCAGCCTGACCTGCCTGGTCAAAGGCTTCTATCCCAGCGACATCGCCGTGGAGTGGGAGAGCAATGGGCAGCCGGAGAACAACTACAAGACCACGCCTCCCGTGCTGGACTCCGACGGCTCCTTCTTCCTCTACAGCAAGCTCACCGTGGACAAGAGCAGGTGGCAGCAGGGGAACGTCTTCTCATGCTCCGTGATGCACGAGGCTCTGCACAACCACTACACGCAGAAGAGCCTCTCCCTGTCTCCGGGTAAATAA

**hATR-5 CAR:**

CCACCATGGATTTTCAGGTGCAGATTTTCAGCTTCCTGCTAATCAGTGCCTCAGTCATAATGTCTGCTCAAGTCCAGCTCCTCGAAAGCGGGGCTGTACTTGCTCGGCCAGGAACCAGTGTAAAAATATCATGTAAGGCGTCTGGATTCAATATAAAGGATTACTATATGCACTGGGTTAAACAAAGGCCCGGCCAAGGATTGGAATGGATAGGAGGCAACGATCCCGCCAACGGTCATTCTATGTATGATCCTAAATTTCAAGGGCGCGTTACAATTACAGCCGATACATCAACTAGCACCGTGTTTATGGAGCTCTCATCCCTCAGATCTGAAGACACCGCCGTATATTATTGTGCTCGAGACAGTGGTTATGCAATGGATTATTGGGGTCAGGGTACTCTTGTCACCGTTAGCAGCGGCGGTGGCGGTTCTGGTGGCGGTGGCTCCGGCGGTGGCGGTTCTGACATTCAGATGACTCAATCTCCTTCAAGTCTTTCTGCTAGTGTAGGTGACAGGGTGACGATAACGTGCAAGGCGAGCCAAGATATAAAGTCTTTCCTGTCTTGGTATCAGCAGAAGCCCGAGAAAGCTCCCAAGAGCTTGATATACTACGCCACGTCTCTCGCAGACGGAGTTCCCTCTCGCTTTTCTGGGTCTGGGTCCGGTACAGACTACACCCTGACGATTAGTTCACTTCAACCGGAAGATTTTGCTACCTACTACTGTCTTCAGCACGGGGAGTCTCCATATACATTTGGGGGTGGGACTAAAGTAGAGATAAAACGCGGTACCGAACAAAAACTCATCTCAGAAGAGGATCTGAATGGGGTCACCTCAGCGCTGAGCAACTCCATCATGTACTTCAGCCACTTCGTGCCGGTCTTCCTGCCAGCGAAGCCCACCACGACGCCAGCGCCGCGACCACCAACACCGGCGCCCACCATCGCGTCGCAGCCCCTGTCCCTGCGCCCAGAGGCGTGCCGGCCAGCGGCGGGGGGCGCAGTGCACACGAGGGGGCTGGACCCCTTTTGGGTGCTGGTGGTGGTTGGTGGAGTCCTGGCTTGCTATAGCTTGCTAGTAACAGTGGCCTTTATTATTTTCTGGGTGAGGAGTAAGAGGAGCAGGCTCCTGCACAGTGACTACATGAACATGACTCCCCGCCGCCCCGGGCCCACCCGCAAGCATTACCAGCCCTATGCCCCACCACGCGACTTCGCAGCCTATCGCTCCCTCGAGAGAGTGAAGTTCAGCAGGAGCGCAGACGCCCCCGCGTACCAGCAGGGCCAGAACCAGCTCTATAACGAGCTCAATCTAGGACGAAGAGAGGAGTACGATGTTTTGGACAAGAGACGTGGCCGGGACCCTGAGATGGGGGGAAAGCCGAGAAGGAAGAACCCTCAGGAAGGCCTGTACAATGAACTGCAGAAAGATAAGATGGCGGAGGCCTACAGTGAGATTGGGATGAAAGGCGAGCGCCGGAGGGGCAAGGGGCACGATGGCCTTTACCAGGGTCTCAGTACAGCCACCAAGGACACCTACGACGCCCTTCACATGCAGGCCCTGCCCCCTCGCTAA

**TF8-5G9 scFvFc**

ATGCGAAGGATGCAACTTCTTCTGCTCATCGCACTTTCCTTGGCGCTGGTGACAAATAGTGAGATTCAACTCCAACAGAGCGGAGCCGAGTTGGTCAGACCTGGCGCACTGGTGAAACTCTCATGTAAGGCCTCCGGTTTTAACATCAAAGATTATTACATGCACTGGGTAAAACAGCGACCGGAACAAGGACTGGAGTGGATAGGGCTTATCGATCCAGAGAATGGTAATACTATCTACGATCCAAAGTTCCAGGGTAAGGCGTCTATTACTGCTGACACTTCTAGTAATACGGCCTACTTGCAACTTTCCTCTCTTACCTCAGAGGACACGGCTGTTTACTACTGCGCTAGGGACAACTCATATTACTTCGACTATTGGGGTCAAGGTACGACGTTGACGGTCTCTTCAGGCGGTGGCGGATCCGGTGGCGGTGGCTCCGGCGGTGGCGGTTCTGATATTAAGATGACACAATCACCCTCTAGTATGTACGCATCATTGGGGGAACGGGTAACTATAACGTGCAAAGCATCCCAGGACATTAGAAAATACCTTAATTGGTATCAACAAAAGCCATGGAAGTCCCCTAAAACATTGATTTATTACGCGACCTCTTTGGCCGATGGCGTCCCAAGTCGGTTTTCTGGAAGTGGCTCTGGTCAGGATTACAGTCTCACAATTTCATCACTGGAGTCCGATGATACCGCAACCTATTATTGCCTCCAACACGGCGAGTCCCCCTATACATTTGGAGGGGGTACTAAACTGGAAATCAATAGGGGTACCGACAAAACTCACACATGCCCACCGTGCCCAGCACCTGAACTCCTGGGGGGACCGTCAGTCTTCCTCTTCCCCCCAAAACCCAAGGACACCCTCATGATCTCCCGGACCCCTGAGGTCACATGCGTGGTGGTGGACGTGAGCCACGAAGACCCTGAGGTCAAGTTCAACTGGTACGTGGACGGCGTGGAGGTGCATAATGCCAAGACAAAGCCGCGGGAGGAGCAGTACAACAGCACGTACCGTGTGGTCAGCGTCCTCACCGTCCTGCACCAGGACTGGCTGAATGGCAAGGAGTACAAGTGCAAGGTCTCCAACAAAGCCCTCCCAGCCCCCATCGAGAAAACCATCTCCAAAGCCAAAGGGCAGCCCCGAGAACCACAGGTGTACACCCTGCCCCCATCCCGGGAGGAGATGACCAAGAACCAGGTCAGCCTGACCTGCCTGGTCAAAGGCTTCTATCCCAGCGACATCGCCGTGGAGTGGGAGAGCAATGGGCAGCCGGAGAACAACTACAAGACCACGCCTCCCGTGCTGGACTCCGACGGCTCCTTCTTCCTCTACAGCAAGCTCACCGTGGACAAGAGCAGGTGGCAGCAGGGGAACGTCTTCTCATGCTCCGTGATGCACGAGGCTCTGCACAACCACTACACGCAGAAGAGCCTCTCCCTGTCTCCGGGTAAATAA

**TF8-5G9 CAR**

ATGGATTTTCAGGTGCAGATTTTCAGCTTCCTGCTAATCAGTGCCTCAGTCATAATGTCTGAGATTCAACTCCAACAGAGCGGAGCCGAGTTGGTCAGACCTGGCGCACTGGTGAAACTCTCATGTAAGGCCTCCGGTTTTAACATCAAAGATTATTACATGCACTGGGTAAAACAGCGACCGGAACAAGGACTGGAGTGGATAGGGCTTATCGATCCAGAGAATGGTAATACTATCTACGATCCAAAGTTCCAGGGTAAGGCGTCTATTACTGCTGACACTTCTAGTAATACGGCCTACTTGCAACTTTCCTCTCTTACCTCAGAGGACACGGCTGTTTACTACTGCGCTAGGGACAACTCATATTACTTCGACTATTGGGGTCAAGGTACGACGTTGACGGTCTCTTCAGGCGGTGGCGGTTCTGGTGGCGGTGGCTCCGGCGGTGGCGGTTCTGATATTAAGATGACACAATCACCCTCTAGTATGTACGCATCATTGGGGGAACGGGTAACTATAACGTGCAAAGCATCCCAGGACATTAGAAAATACCTTAATTGGTATCAACAAAAGCCATGGAAGTCCCCTAAAACATTGATTTATTACGCGACCTCTTTGGCCGATGGCGTCCCAAGTCGGTTTTCTGGAAGTGGCTCTGGTCAGGATTACAGTCTCACAATTTCATCACTGGAGTCCGATGATACCGCAACCTATTATTGCCTCCAACACGGCGAGTCCCCCTATACATTTGGAGGGGGTACTAAACTGGAAATCAATAGGGAACAAAAACTCATCTCAGAAGAGGATCTGAATGGGGTCACCGTCTCTTCAGCGCTGAGCAACTCCATCATGTACTTCAGCCACTTCGTGCCGGTCTTCCTGCCAGCGAAGCCCACCACGACGCCAGCGCCGCGACCACCAACACCGGCGCCCACCATCGCGTCGCAGCCCCTGTCCCTGCGCCCAGAGGCGTGCCGGCCAGCGGCGGGGGGCGCAGTGCACACGAGGGGGCTGGACCCCTTTTGGGTGCTGGTGGTGGTTGGTGGAGTCCTGGCTTGCTATAGCTTGCTAGTAACAGTGGCCTTTATTATTTTCTGGGTGAGGAGTAAGAGGAGCAGGCTCCTGCACAGTGACTACATGAACATGACTCCCCGCCGCCCCGGGCCCACCCGCAAGCATTACCAGCCCTATGCCCCACCACGCGACTTCGCAGCCTATCGCTCCCTCGAGAGAGTGAAGTTCAGCAGGAGCGCAGACGCCCCCGCGTACCAGCAGGGCCAGAACCAGCTCTATAACGAGCTCAATCTAGGACGAAGAGAGGAGTACGATGTTTTGGACAAGAGACGTGGCCGGGACCCTGAGATGGGGGGAAAGCCGAGAAGGAAGAACCCTCAGGAAGGCCTGTACAATGAACTGCAGAAAGATAAGATGGCGGAGGCCTACAGTGAGATTGGGATGAAAGGCGAGCGCCGGAGGGGCAAGGGGCACGATGGCCTTTACCAGGGTCTCAGTACAGCCACCAAGGACACCTACGACGCCCTTCACATGCAGGCCCTGCCCCCTCGCTAA

**TF8-5G9-OKT3 BiTE**

ATGGATTTTCAGGTGCAGATTTTCAGCTTCCTGCTAATCAGTGCCTCAGTCATAATGTCTGATATTAAGATGACACAATCACCCTCTAGTATGTACGCATCATTGGGGGAACGGGTAACTATAACGTGCAAAGCATCCCAGGACATTAGAAAATACCTTAATTGGTATCAACAAAAGCCCTGGAAGTCCCCTAAAACATTGATTTATTACGCGACCTCTTTGGCCGATGGCGTCCCAAGTCGGTTTTCTGGAAGTGGCTCTGGTCAGGATTACAGTCTCACAATTTCATCACTGGAGTCCGATGATACCGCAACCTATTATTGCCTCCAACACGGCGAGTCCCCCTATACATTTGGAGGGGGTACTAAACTGGAAATCAATAGGGGCGGTGGCGGTTCTGGTGGCGGTGGCTCCGGCGGTGGCGGTTCTGAGATTCAACTCCAACAGAGCGGAGCCGAGTTGGTCAGACCTGGCGCACTGGTGAAACTCTCATGTAAGGCCTCCGGTTTTAACATCAAAGATTATTACATGCACTGGGTAAAACAGCGACCGGAACAAGGACTGGAGTGGATAGGGCTTATCGACCCAGAGAATGGTAATACTATCTACGATCCAAAGTTCCAGGGTAAGGCGTCTATTACTGCTGACACTTCTAGTAATACGGCCTACTTGCAACTTTCCTCTCTTACCTCAGAGGACACGGCTGTTTACTACTGCGCTAGGGACAACTCATATTACTTCGACTATTGGGGTCAAGGTACGACGTTGACGGTCTCTTCAGGCGGAGGATCCCAGGTCCAGCTGCAGCAGTCTGGGGCTGAACTGGCAAGACCTGGGGCCTCAGTGAAGATGTCCTGCAAGGCTTCTGGCTACACCTTTACTAGGTACACGATGCACTGGGTAAAACAGAGGCCTGGACAGGGTCTGGAATGGATTGGATACATTAATCCTAGCCGTGGTTATACTAATTACAATCAGAAGTTCAAGGACAAGGCCACATTGACTACAGACAAATCCTCCAGCACAGCCTACATGCAACTGAGCAGCCTGACATCTGAGGACTCTGCAGTCTATTACTGTGCAAGATATTATGATGATCATTACTGCCTTGACTACTGGGGCCAAGGCACCACTCTCACAGTCTCCTCAGGTGGGTCAGGGGGCTCTGGAGGTTCAGGCGGGAGTGGGGGTCAAATTGTTCTCACCCAGTCTCCAGCAATCATGTCTGCATCTCCAGGGGAGAAGGTCACCATGACCTGCAGTGCCAGCTCAAGTGTAAGTTACATGAACTGGTACCAGCAGAAGTCAGGCACCTCCCCCAAAAGATGGATTTATGACACATCCAAACTGGCTTCTGGAGTCCCTGCTCACTTCAGGGGCAGTGGGTCTGGGACCTCTTACTCTCTCACAATCAGCGGCATGGAGGCTGAAGATGCTGCCACTTATTACTGCCAGCAGTGGAGTAGTAACCCATTCACGTTCGGCTCGGGGACAAAGTTGGAAATAAACCGGGAACAAAAACTCATCTCAGAAGAGGATCTGAATGGGCATCACCACCATCACCATCATCACTAA

**TF-Fc**

ATGCGAAGGATGCAACTTCTTCTGCTCATCGCACTTTCCTTGGCGCTGGTGACAAATAGTACTACAAATACTGTGGCAGCATATAATTTAACTTGGAAATCAACTAATTTCAAGACAATTTTGGAGTGGGAACCCAAACCCGTCAATCAAGTCTACACTGTTCAAATAAGCACTAAGTCAGGAGATTGGAAAAGCAAATGCTTTTACACAACAGACACAGAGTGTGACCTCACCGACGAGATTGTGAAGGATGTGAAGCAGACGTACTTGGCACGGGTCTTCTCCTACCCGGCAGGGAATGTGGAGAGCACCGGTTCTGCTGGGGAGCCTCTGTATGAGAACTCCCCAGAGTTCACACCTTACCTGGAGACAAACCTCGGACAGCCAACAATTCAGAGTTTTGAACAGGTGGGAACAAAAGTGAATGTGACCGTAGAAGATGAACGGACTTTAGTCAGAAGGAACAACACTTTCCTAAGCCTCCGGGATGTTTTTGGCAAGGACTTAATTTATACACTTTATTATTGGAAATCTTCAAGTTCAGGAAAGAAAACAGCCAAAACAAACACTAATGAGTTTTTGATTGATGTGGATAAAGGAGAAAACTACTGTTTCAGTGTTCAAGCAGTGATTCCCTCCCGAACAGTTAACCGGAAGAGTACAGACAGCCCGGTAGAGTGTATGGGCCAGGAGAAAGGGGAGTTCAGAGAAGGTACCGACAAAACTCACACATGCCCACCGTGCCCAGCACCTGAACTCCTGGGGGGACCGTCAGTCTTCCTCTTCCCCCCAAAACCCAAGGACACCCTCATGATCTCCCGGACCCCTGAGGTCACATGCGTGGTGGTGGACGTGAGCCACGAAGACCCTGAGGTCAAGTTCAACTGGTACGTGGACGGCGTGGAGGTGCATAATGCCAAGACAAAGCCGCGGGAGGAGCAGTACAACAGCACGTACCGTGTGGTCAGCGTCCTCACCGTCCTGCACCAGGACTGGCTGAATGGCAAGGAGTACAAGTGCAAGGTCTCCAACAAAGCCCTCCCAGCCCCCATCGAGAAAACCATCTCCAAAGCCAAAGGGCAGCCCCGAGAACCACAGGTGTACACCCTGCCCCCATCCCGGGAGGAGATGACCAAGAACCAGGTCAGCCTGACCTGCCTGGTCAAAGGCTTCTATCCCAGCGACATCGCCGTGGAGTGGGAGAGCAATGGGCAGCCGGAGAACAACTACAAGACCACGCCTCCCGTGCTGGACTCCGACGGCTCCTTCTTCCTCTACAGCAAGCTCACCGTGGACAAGAGCAGGTGGCAGCAGGGGAACGTCTTCTCATGCTCCGTGATGCACGAGGCTCTGCACAACCACTACACGCAGAAGAGCCTCTCCCTGTCTCCGGGTAAACATCACCACCATCACCATCATCACTGA
